# Prostate artery embolization: increasing self-referrals and awareness of treatment options

**DOI:** 10.1186/s42155-021-00251-5

**Published:** 2021-08-23

**Authors:** Himanshu Sharma, Samuel Z. Maron, Ardeshir R. Rastinehad, Aaron M. Fischman

**Affiliations:** 1 Icahn School of Medicine at Mount Sinai, Mount Sinai Hospital, 10029 New York, NY USA; 2 The Smith Institute for Urology, Northwell Health, Lenox Hill Hospital, 245 E 54th St., 320 Central Park West, Apt 2A, NY 10022 New York, USA

## Introduction

Prostatic artery embolization (PAE) is a minimally invasive therapy performed as an elective outpatient procedure for lower urinary tract symptoms (LUTS) secondary to benign prostatic hyperplasia (BPH). Multiple studies have demonstrated the efficacy of PAE in reducing LUTS over the medium and long term (Pisco et al. 2016; Carnevale et al. 2020). PAE is transitioning from the research setting to implementation as a routine clinical procedure (Young and Golzarian 2019). The number of patients treated with PAE has grown in recent years, but the current literature has not well characterized awareness of PAE as a treatment option among patients. As PAE becomes more widespread, patients may not want to wait for a referral from a urologist or primary care physician, and instead self-refer to interventional radiology (IR) directly. IR practices in turn may need to engage with the patient community more directly and increase their online presence.

## Methods

PAE cases from January 2015 until August 2020 at two hospitals in a tertiary academic health system were queried. Data from all consecutive PAE patients (*n* = 215) for any indication were included in this study. The patient referral source was collected by review of electronic medical records.

## Results

The most common source of referral was urology (*n* = 104, 48 %), followed by self-referral (*n* = 64, 30 %), internal medicine/primary care provider (*n* = 23, 11 %), inpatient consult (*n* = 14, 6 %), emergency department (*n* = 5, 2 %), external interventional radiology (*n* = 4, 2 %), and oncology (*n* = 1, 1 %). These results are summarized in Table 1.

**Table 1:**
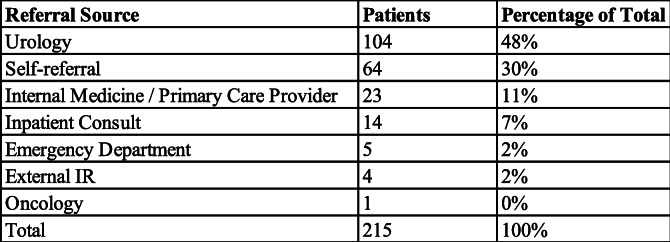
Total referrals by source 2015 - 2020

**Figure 1:**
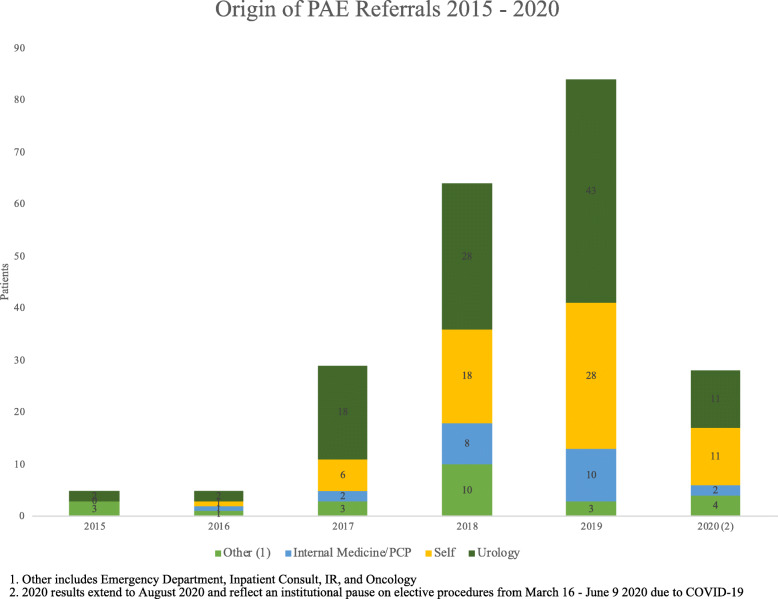
Fig. 1

Referral sources were calculated by year and results are summarized in Table 2. While the sample sizes in 2015 and 2016 are small, the number of self-referred patients grew substantially from 6 patients in 2017 to 28 patients in 2019 (Fig. 1). There were 11 self-referrals in 2020 although the data only extends to August and is further limited by the institutional pause on elective procedures from March 16, 2020 to June 9, 2020 due to COVID-19. The proportion of self-referred patients also increased each year from the prior year. The proportion of self-referrals in 2015 was 0 % (*n* = 5), 20 % in 2016 (*n* = 5), 21 % in 2017 (*n* = 29), 29 % in 2018 (*n* = 64), 35 % in 2019 (*n* = 84), and 39 % in 2020 (*n* = 28). Urology is the largest referral source, but the share of urology referrals has decreased from 62 % to 2017 to 39 % in 2020. In 2020, the number of self-referrals was equal to urology referrals. Referrals from internal medicine/primary care provider increased from 7 % of total referrals in 2017 to 12 % in 2019. The distribution of referrals from urology, self-referral, primary care providers, and other sources between 2015 and 2020 is summarized in Fig. 1.

**Table 2:**
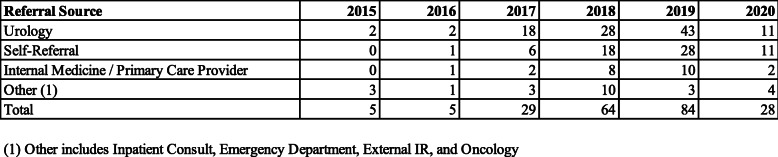
Referral sources by year from 2015 - 2020

## Discussion

The results of our retrospective review demonstrate the increasing popularity of PAE among patients. Excluding small samples in 2015 and 2016, PAE volume increased by 121 % from 2017 to 2018 and 31 % from 2018 to 2019. Our findings also demonstrate that while urology is the largest referral source, the share of urology referrals has decreased from 2017 to 2020. An increasing number of patients self-refer for PAE. Between 2017 and 2020, self-referral has grown from 21 % of PAE volume to 39 %.

Further study of patients who self-referred to determine the method through which they learned about PAE, such as word-of-mouth, advertising, media, or other sources, could allow IR practices to reach more patients directly. While our results indicate a growing appetite for PAE among our patient population, to our knowledge there have been no large studies assessing patient understanding of PAE. As the generalizability of the present study is limited by its single-system design, future work examining PAE referral and volume at the regional and national level will be useful in assessing broader patient knowledge of and access to PAE. Patients have a wide range of options for treatment for LUTS secondary to BPH, ranging from conservative medical management, to minimally invasive options including PAE, to more invasive procedures such as transurethral prostate resection, Urolift, Rezum, and prostatectomy. Greater understanding of the patients who self-referred to PAE may help interventional radiologists assess the methods by which they can educate BPH patients about minimally invasive alternatives.

In addition to further study of patient knowledge of PAE, assessment of awareness of PAE among primary care providers could be useful. Internal medicine/primary care provider referrals for PAE grew from 2 to 2017 to 10 in 2019, but comprised a smaller share of total referrals in 2019 (12 %) than urology (51 %) and self-referrals (33 %). Educating internal medicine, geriatrics, and family medicine departments on PAE as a treatment option for BPH could present an opportunity to reach more patients and cultivate another referral source.

As seen with the relatively low penetration of uterine fibroid embolization compared to hysterectomy, patient awareness of IR procedures can be concerningly low despite establishment of safety and efficacy (Makris et al. 2020). Further study of public awareness of PAE is crucial in spreading access to all patients who might benefit from the procedure.

## Conclusions

These preliminary findings suggest that urology continues to be the most prevalent source of PAE referrals, with an increasing proportion of self-referrals. The growing number of self-referrals suggests an increasing awareness of PAE as a treatment option in the patient community. Further study of patient awareness and understanding of PAE could be useful in increasing access to the procedure.

## Data Availability

The datasets generated and/or analyzed during the current study are not publicly available due to protected patient health information but are available from the corresponding author on reasonable request.

## Abbreviations

PAE: Prostate Artery Embolization
LUTS: Lower Urinary Tract Symptoms
BPH: Benign Prostatic Hyperplasia
IR: Interventional Radiology
